# Ingrowing Toe-Nail

**Published:** 1884-06

**Authors:** 


### INGROWING TOE-NAIL.

BY

THE EDITOR.

---

THE treatment of Ingrowing Toe-nail is so unsatisfactory as to be almost an opprobrium to our art. And yet, of the minor surgical ailments few have been honoured with more attention. Such men as Paré, Dupuytren, Desault, Sir Astley Cooper, and Colles have deemed the subject worthy of their special study; while a host of others, too numerous to mention, have at various times recommended plans of cure as diverse almost as their names.

Amidst all this diversity there appear to stand out only two facts on which all are agreed. The first is, that the complaint is caused by the wearing of tight boots; the second, that it is not ingrowing of the toe-nail at all, but overgrowing of the toe-flesh. With reference to the first point, it seems to have struck no one as peculiar that the leaving off tight boots is by no means a cure for the malady. *Sublata causa, tollitur effectus* ought to hold good here as elsewhere. And if we say, with greater show of reason, that it is simply an outcome of the civilized habit of wearing coverings for the feet of any sort, which prevent the toes from spreading out as we walk, and demand that to give a cure we must walk bare-footed, we might still require proof that savages do not have ingrowing toe-nail. I strongly suspect that they do. As to the second point of agreement, that it is rather overgrowing of the flesh than ingrowing of the nail, we might here also ask why, if these writers believed this, they so diligently attack the innocuous nail? True, if there were no offending toe-nail, there would be no recalcitrant sore flesh, and the prompt annihilation of one of the offenders would extinguish the disagreement. But the nail grows again, and the flesh rebels as before, and the trouble is soon as bad as ever.

In the following account I have systematised what I know of the causation of the complaint, and what I have found to be the best means of treating it. And let me say at the outset, that as I believe the causes to be various and distinct, so am I convinced that success in treatment depends on an accurate appreciation of these. One treatment for ingrowing toe-nail is as unscientific as one treatment for ulcerated legs.

Definition.—A chronic, painful, traumatic inflammation of the tissues at the margin of a toe-nail. The inflammation is usually attended with the formation of granulations and with suppuration; and it is nearly always of the great toe-nail, usually on its outer side. There is a form of so-called ingrowing toe-nail which is not attended with suppuration, but is dependent on an accumulation of epidermic scales between the nail and the flesh; and, very rarely, the disease may exist in one or other of the lesser toes.

Causation.—In civilized countries we must always recognize the element of compression, or at least prevention of expansion, inside a boot. It is perfectly conceivable that the condition might exist in individuals who never wear boots; but for practical purposes we must take the boots for granted. They are a constant concomitant; and if not a prime, are probably a contributing cause. It is, however, a cause which we cannot remove. We must treat the toe inside the boots. Indeed, the patient will probably have removed the cause long before we see him. He must crucify the flesh even more than the monks who walked on hard peas, who will wear tight boots over an ingrowing nail. Looking beyond the boots, we find that the causes may be arranged as Intrinsic, or depending on peculiarities in the toe or nail; and Extrinsic, or dependent on the direction of the toes or the condition of outlying structures in the foot.

I.—INTRINSIC, *i.e.*, in the nail, or in the surrounding tissues, or in both.

1. *In the nail.* In some people the nails in the fingers and the toes—and I have noticed that the peculiarity is usually coincident—are convex or arched, and dip deeply into the surrounding flesh. In such cases, in paring the nail of the great toe it is difficult to carry the knife or scissors completely round, and thus there is frequently left behind a small spicule or pointed piece, which readily insinuates itself into the neighbouring flesh. Matters are sometimes made worse by pulling at this piece, "tearing it to the quick." The flesh swells and conceals this small piece of outlying nail; it is overlooked, sets up irritation, is concealed in the swollen flesh, and the condition is developed.

2. *In the flesh.* Some people have a redundance of flesh in their toes, and their fingers as well. They are said to have "flabby" fingers. In these the flesh overlaps the nail, and in the foot the confinement of the boot, added to the soddening perspiration under the overlapping flesh, readily starts the condition. Once started it continues, and suppuration along the margin of such a toe may continue for years. Fortunately, it is the least painful and most easily treated of all the varieties.

3. *In both nail and flesh.* The existence of both the above conditions—an arched toe-nail, and an excess of soft tissue—will frequently be found associated with the malady. Alone, or in combination with extrinsic causes, this double condition, with the mere wearing of boots, is almost enough to cause the complaint. In this case also it is not likely to be severe.

II.—Extrinsic, or from causes lying outside the nail and its surrounding tissues.

1. *Flattening of the arch of the foot.* That flat-foot may be a cause, and a frequent and important cause, of in-growing nail is not a generally recognised fact in surgery. It will be well, therefore, shortly to explain the manner of its production.

Part of the explanation is given in the previous number of this *Journal*, where Professor Ogston describes the causation of flat-foot. The completion of the account lies in the attempt of the point of the great toe to become the anterior pillar of the arch of the foot. When the natural arch is destroyed the anterior pillar, the ball of the great toe, atrophies, and the patient rests the weight of the body on the whole of the flattened sole. At the end of the step, when the knee is flexed, the weight is transferred to the anterior portion of the foot. This is naturally the pad at the root of the toes, particularly of the great toe; but when the plantar ligaments are relaxed and perhaps painful, this support is not available. Recource is then had to the only available help, the only tendon which passes from the top of the arch of the foot to its anterior extremity, viz., that of the flexor longus pollicis. By its contraction the tip of the great toe is brought to the ground, and acts as a substitute for the natural pier. But constant use of the toe in this wise induces hypertrophy of its tissue, and consequent overlapping of the toe-nail. By easily understood stages this hypertrophy becomes irritation, inflammation and suppuration where the flesh is crowded over the edges of the nail, and we thus get the condition fully developed.

It is simple flat-foot, pes planus, and not splay-foot, or pes valgus, which is most likely to start the mischief. If splay-foot causes it, it is more by everting the toe from pressure on its inside in walking, and so squeezing it against the second toe. And it has seemed to me that not the worst cases of flat-foot—those which require operation—but the moderate cases, which require no special treatment for the flattening, are chiefly associated with ingrowing nail. Three cases of flat-foot, on which I had to operate, had no sign of ingrowing nail.

Flat-foot, in varying degrees, I believe to be the most important cause of ingrowing toe-nail, and all the more so that the ordinary modes of treatment are futile to cure it.

2. *Eversion of the great toe.*—Though forcing of the toe outwards by wearing pointed boots, or the dislocation attendant on chronic rheumatic arthritis, or on bunion, might be included as causes under this head, yet I believe its production will more frequently be found to depend either on a habit of walking with the limb much rotated outwards, or on a congenital deflection of the toe itself. This too close proximity may merge into a passing beyond, and then we have the second toe, perhaps with the third, overriding the great toe, and evidently causing the complaint.

I have had to treat this condition in three members of one family, in whom the tendency was well known to exist, and was sought to be minimised by the wearing of broad-toed boots.

3. *Inversion of the lesser toes.*—In this case the same result as the preceding is produced by a deviation inwards of the second and third toes. How it is produced I do not know. I have seen it in a boy whose father told me he suffered from the same thing in his youth, and that another son had it in less degree. In two Infirmary patients—also boys—it was not a family peculiarity. In all there was suppuration and pain in walking, though not as severe as in other varieties.

TREATMENT.—From what has been said it will be seen that the scientific position of ingrowing toe-nail is chiefly as a symptom, the most prominent and distressing symptom, of several other conditions. By removing these causative conditions we do away with the common effect—the ingrowing nail; and we will be successful according to the accuracy with which the cause is diagnosed and counteracted.

I.—1. Where the cause is intrinsic and resident in the nail alone, it may usually be remedied by careful attention to the "toilet" of the nail, using a knife rather than a scissors, and cutting from behind forwards obliquely, so as to give the nail a pointed shape. By this means the leaving behind of sharp portions at the margin which are insinuated into the flesh is rendered less likely. If the granulations are exuberant I would recommend the application of a crystal or two of chromic acid, which leaves a hard dry scab, under which the sore heals kindly. Careful trimming of the nail will usually ward off the complaint in the future.

2. Where the cause lies in a superabundance of flesh in the toe, a condition which is usually accompanied with thin, tender skin, which perspires and chafes readily, I believe the best plan to be: first, the application of chromic acid, if necessary, and thereafter pressure either by strapping or by elastic. Every night the affected toe is to be surrounded tightly from the tip upwards by thin strips of adhesive plaster taken out of boiling water. This may be removed in the morning and replaced by an India-rubber cap, such as is worn over a sore finger during a *post mortem* examination. The toe is thus rendered and kept anæmic by compression; congestion is removed, and the tissues get more firm and resisting in the course of a few months.

In such cases I have sometimes noticed that the feet perspire freely, and then the wearing of fine worsted socks, the nightly use of a foot-bath into which enough sulphuric acid has been poured to make the skin tingle, and sprinkling some powdered boracic acid by means of a pepper-box over the foot every morning, will expedite he cure.

3. When there is a combination of malformed nail and overgrowth of flesh, a judicious combination of the methods just described will probably effect a cure. Here, if anywhere, a scraping of the nail, making it thin and yielding, ought to do good; but I am doubtful of the utility of this procedure. The nail is too firmly bound down to the matrix to yield much to lateral pressure; and constant scraping, I think, has a tendency to develop an irritative hypertrophy of the nail itself. If all these or similar plans fail, there is nothing for it but removal of the nail in a manner to be described presently.

II.—1. Of extrinsic causes by far the most important is flattening of the arch of the foot; and unless this cause is clearly recognised and successfully met our treatment will almost certainly fail. If the explanation just given be admitted, the indication is clearly either to do away with the necessity for the compensatory action of the flexor longus pollicis, or to raise the toe above the possibility of its coming to harm—*i.e.*, to *force* the ball of the great toe to become the anterior support of the arch. To restore the arch of the foot, probably the most scientific treatment would be to make the patient recline on his back for some weeks, and permit the stretched plantar ligaments to regain their tone. A pathological excess of such a proceeding may be seen in what I am in the habit of describing as "postural club foot," so frequently seen after prolonged rest in the supine position, as for hip-joint disease or fractured thigh, or such like, where the foot is not supported. The temporary use of a plaster of Paris casing might be suggested; but this proceeding would probably, by pushing up the plantar ligaments, prevent them becoming shorter. In actual practice it will be found a very efficient plan to wear a small pad of several thicknesses of chamois leather or flannel under the ball of the great toe. This pad may be put on every morning, and retained in position by a collar of thread or elastic carried round the root of the great toe. The toe, thus elevated beyond the reach of harm and relieved from its illegitimate labour, soon regains its normal condition. After a few months the pad may be gradually given up; and with care the condition need not recur.

This treatment first suggested itself to me some years ago, after a careful study of the case of a friend who had for years suffered much from ingrowing toe-nail. He had had the nail twice removed, and had undergone with praiseworthy patience every conceivable form of palliative treatment—all without relief. The use of a pad for six months, and some rest to the instep, permanently cured the complaint.

2. When the cause is eversion of the great toe, from whatever cause arising, the treatment is by no means easy. The use inside the boot of springs, such as Biggs' apparatus, to keep the toe inverted, is both difficult and unsatisfactory. What I have found most satisfactory is a pad between the great and second toes, stopping short of the sore part. The pad may be constructed of several layers of flannel or chamois, and is kept in position by two collars round the root of the great and second toes respectively.

3. I have seen only three cases of the second and third toes overlapping the first and causing ingrowing of its nail. In one case several members of the family had suffered from the same complaint. In these cases the condition was easily remedied by wearing a double band of tape, so arranged as to keep the two offending toes turned outwards and pushed downwards. The tape was fixed in a loop round the fourth toe, passed double over the second and third toes, and then surrounded the great toe. The little apparatus is easily made by the patient.

So much for the scientific treatment of the complaint. But there is a class of cases, chiefly among hospital patients, in which imperfect intelligence and want of cleanliness nullify our efforts. The patient is probably the breadwinner of the family, and he is driven out of all patience by the constant worry of his petty complaint. He wants to get well at once and permanently, and will be trifled with no longer with pressure by cork, tin, or lead, or with burning or paring or scraping. Such patients have usually flat-foot; but the endless worry of the morning pad is beyond their endurance. For all these I remove the matrix as well as the nail, and scrape the periosteum off the bone. The operation is a simple one, and by the exercise of a little dexterity may be done on both feet while the patient is under the influence of nitrous oxide gas. The knife grazing the bone is carried rapidly round the flesh on the right side of the nail, and by a change of the same movement passes under the nail down to the bone, and lifts away nail, matrix, and suppurating flesh. A piece of boracic lint is wrapped tightly round the toe, and need not be removed for a week. In the meantime the patient may get about. At the end of the week the sore will be smaller than the nail removed, for the healthy tissues have been pressed inwards over the sore. In three weeks the wound is cicatrised over; and most likely in a few weeks more a stunted nail is developed, like that usually seen on the fifth toe, from which no trouble ever arises.

The sore from this operation heals as quickly as that from simple avulsion of the toe-nail; and there is no young and tender nail to be guided in its growth, and perhaps cause the old trouble in its maturity. This simple cicatrisation is altogether a more rapid and less painful process than the growing of a young nail.

If the patient is not anxious to have a handsome nail on his toe, I never hesitate to let him have this mode of cure. A toe-nail is not much loss. In human beings, especially in the booted, and more particularly in the large mass of humanity who are not and never will be sculptors' models, the toe-nail is neither a useful nor an ornamental appendage. We do not scratch with it; and as a thing of beauty the thickened, stunted, brown epidermic growth usually seen on the great toe of civilized beings can easily be dispensed with. The loss of it, at its best, can never be a great one; and when it is ingrowing its loss is a gain.

As a plan of treatment I believe that mere avulsion of the toe-nail ought to be abolished from surgery. It gives much discomfort during the growth of the young nail, does not attack the true cause of the mischief, and is most uncertain as a mode of cure. The toe-nail is useless; a firm cicatrix is innocuous, and not more unsightly than the ordinary toe-nail of the adult. The removal of the matrix and the scraping of the bone is certain to cure the very worst case of the disease; and therefore, on these grounds, I confidently recommend the proceeding. Scientifically, its position is as a last resort; practically, it may often be the patient's first relief, and the relief will be permanent.

